# Synchronous malignant rhabdoid tumor of the kidney and adrenal neuroblastoma in an infant treated with proton beam therapy: a case report and literature review

**DOI:** 10.3389/fonc.2025.1699028

**Published:** 2026-02-05

**Authors:** Wei Han, Huaida Teng, Junlei Shi, Nan Zhang, Jargal Tumendemberel, Ping Lu, Jie Wang, Jie Zheng, Xinan Liu, Deguang Meng, Shosei Shimizu

**Affiliations:** 1 Department of Pediatric Oncology, Beijing Children’s Hospital, Capital Medical University, Beijing, China; 2 Department of Pediatric Surgery, Beijing Children’s Hospital Baoding Hospital, Capital Medical University, Baoding, China; 3 Department of Pathology, Beijing Children’s Hospital, Capital Medical University, National Center for Children’s Health, Beijing, China; 4 Pediatric Proton Beam Therapy Center, Hebei Yizhou Cancer Hospital, Zhuozhou, China; 5 Department of Pathology, Hebei Yizhou Cancer Hospital, Zhuozhou, China; 6 Department of Radiotherapy Physics and Technology, Hebei Yizhou Cancer Hospital, Zhuozhou, China; 7 Nuclear Medicine Department/Pediatric Proton Beam Therapy Center, Hebei Yizhou Cancer Hospital, Zhuozhou, China; 8 Department of Radiation Oncology, University of Tsukuba, Tsukuba, Japan

**Keywords:** case report, infant, neuroblastoma malignant rhabdoid tumor, proton beam therapy, radiotherapy, IMRT (intensity-modulated radiation therapy), pencil beam scanning proton beam therapy

## Abstract

This case report and literature review presents a 4-month-old infant with a synchronous malignant rhabdoid tumor of the kidney and adrenal neuroblastoma, an exceptionally rare combination without established treatment guidelines. Following surgical resection, proton beam therapy (14 Gy in 10 fractions) was administered to the abdominal–pelvic tumor bed with stringent organ-sparing objectives. Contemporary literature supports proton beam therapy’s (PBT’s) dosimetric advantages for organ preservation in pediatric abdominal malignancies. At 1-year follow-up, the patient remained disease-free, with preserved renal function and normal skeletal development, supporting PBT’s role in minimizing late effects while achieving local control in infant malignancies when conventional photon radiotherapy would pose substantial developmental risks.

## Introduction

Malignant rhabdoid tumor is an extremely rare neoplasm, and no established treatment protocol exists. Multimodal therapy combining surgery, chemotherapy, and radiotherapy is typically considered. Notably, the literature emphasizes that early diagnosis and multidisciplinary treatment are crucial for improving survival in this disease (1).

Pediatric cancers are inherently rare, and synchronous multiple malignancies in children pose additional diagnostic and therapeutic challenges. As highlighted in the Children’s Oncology Group review on rare tumors (2), their extremely low incidence makes early clinical suspicion difficult, and the nonspecific nature of initial symptoms often leads to diagnostic delay. Radiologic findings are generally limited, and definitive histopathological diagnosis requires advanced techniques such as immunohistochemistry and molecular testing; however, the scarcity of specialized pediatric pathologists frequently necessitates external expert review. Therapeutically, the lack of large-scale clinical trials hampers the establishment of standardized treatment protocols, leading to substantial variation in management across institutions. In resource-limited settings, restricted access to high-quality imaging, molecular diagnostics, and advanced modalities such as proton beam therapy may further contribute to delays and reduced treatment intensity.

Radiotherapy plays a critical role in local control of pediatric solid tumors, with utilization rates in pediatric neuroblastoma estimated at approximately 64%, a figure that underscores its clinical importance (3). Nevertheless, its application in very young children, particularly those under 3 years old, raises substantial concerns about long-term developmental and late adverse effects such as endocrinopathy, neurocognitive impairment, growth and musculoskeletal disorders, and secondary malignancies (4). High-precision radiotherapy techniques, including IMRT and proton beam therapy, are increasingly adopted in the management of neuroblastoma to minimize radiation exposure to organs at risk (OARs). Proton beam therapy, by exploiting the Bragg peak effect, markedly reduces exit dose and thereby lowers the risk of radiation-associated toxicities (5). A 2025 review confirmed that proton beam therapy (PBT) reduces the risks of cognitive, neuroendocrine, growth, and musculoskeletal disorders, as well as cardiovascular dysfunction, compared to conventional photon therapy (6). In cases involving high-grade tumors or an elevated risk of recurrence, radiotherapy may still be unavoidable regardless of patient age, owing to the imperative for effective local disease control (3).

## Patient information and clinical findings

A 4-month-old girl presented after her parents incidentally palpated a mass in her left abdomen. Imaging studies revealed a space-occupying lesion in the left kidney and a mass in the left adrenal gland. She underwent one-stage radical surgery consisting of a left nephrectomy, a left partial adrenalectomy, and an abdominal lymph node dissection. Gross total resection of both tumors was attempted.

## Timeline

**Figure 1:**
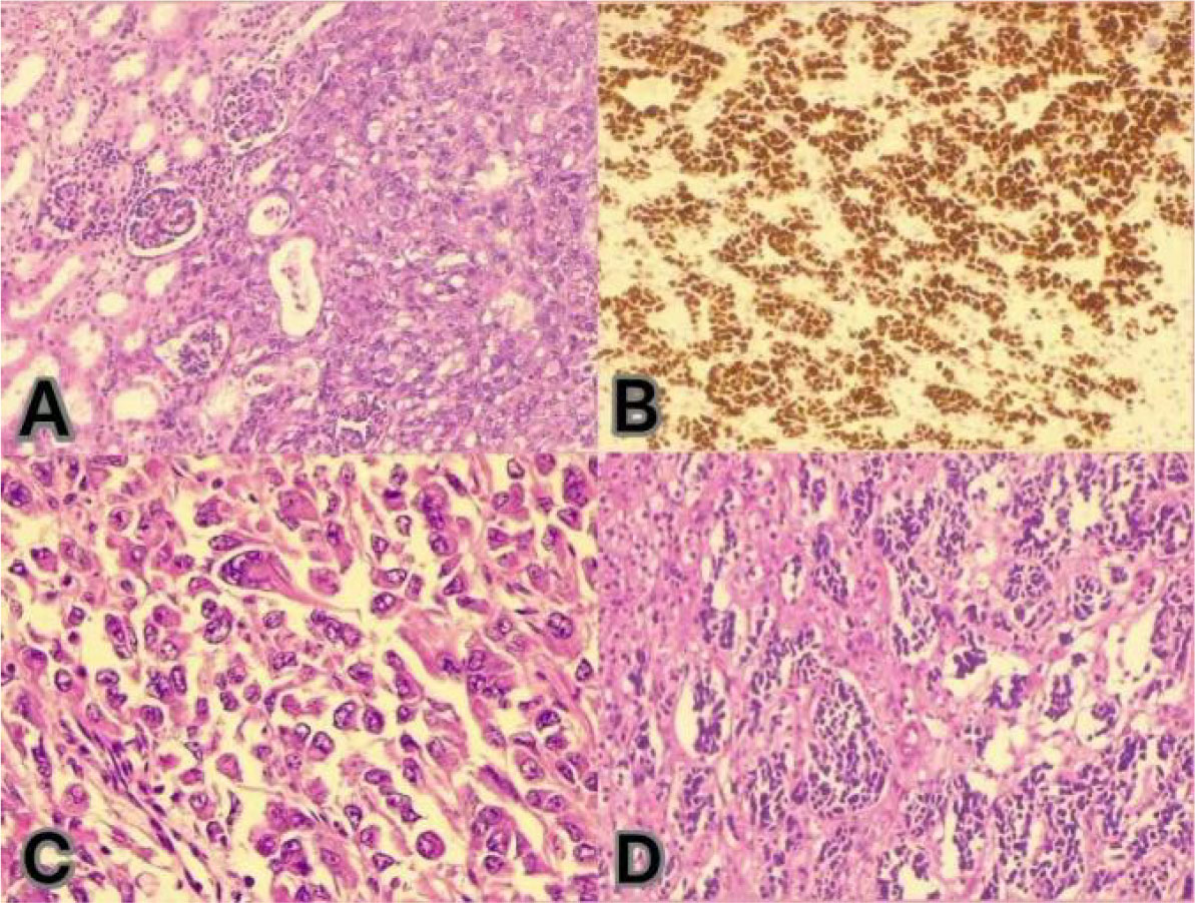
**(A)** Immunohistochemistry for INI-1 (SMARCB1) shows a complete loss of nuclear expression in the tumor cells, confirming the diagnosis of malignant rhabdoid tumor (MRT). **(B, D)** Hematoxylin and eosin (H&E) staining of the renal tumor demonstrates classic features of MRT, including sheets of discohesive cells with eccentric nuclei, prominent nucleoli, and hyaline cytoplasmic inclusions. **(C)** H&E staining of the adrenal tumor reveals a small round blue cell tumor with neuroblastic differentiation.

Histopathological examination confirmed two independent primary tumors (**Figure 1**), the characteristics of which are summarized in **Tables 1**, **2**.

**Table 1:** Molecular and genetic testing.

Test category	Method	Key findings	Clinical interpretation
Somatic mutation panel (123 genes)	NGS	CDK12 c.1912G>A (p.G638R) (VUS, 48.49%) No pathogenic mutations in ALK, BRCA1/2, BRAF, RAS, EGFR, and NTRK	No actionable mutations identified; VUS requires monitoring but does not guide therapy
Immunotherapy biomarkers	MMR gene analysis	MMR normal	No evidence of hypermutation; immunotherapy response unlikely
Chemotherapy sensitivity markers	Gene-drug sensitivity prediction	Potential sensitivity to irinotecan, vincristine, and cyclophosphamide	Suggests standard agents may be effective
Germline hereditary cancer panel	NGS panel	No pathogenic germline variants	No hereditary cancer predisposition identified
Copy number variation (CNV)	CNV analysis	No MYCN amplification	Favorable risk features; no high-risk cytogenetic markers
		No 1p deletion	
		No 11q deletion	
		No high-risk segmental aberrations	

There was a loss of SMARCB1 (INI1), whereas no loss of SMARCA4 was identified.

NGS, Next-Generation Sequencing; VUS, Variant of Uncertain Significance.

**Table 2:** Patient and tumor characteristics (n = 1).

Parameter	MRT (left kidney)	Neuroblastoma (left adrenal)
Age at diagnosis	4 months	4 months
Histology	Malignant rhabdoid tumor	Undifferentiated neuroblastoma
Immunohistochemistry	INI1−, SMARCA4+, Ki-67 ~90%	PHOX2B+, synaptophysin+, Ki-67 ~80%
Stage	Stage III	Stage III
Metastases	11/14 regional LN positive	LN positive
Genetic/molecular findings	There was a loss of SMARCB1 (INI1), whereas no loss of SMARCA4 was identified.	

MRT, malignant rhabdoid tumor; LN, Lymph Node.

The patient received a total of eight chemotherapy cycles. One cycle was administered immediately after surgery, followed by seven cycles after the completion of radiotherapy. The chemotherapy regimen consisted of four cycles of vincristine, doxorubicin, and cyclophosphamide (VDC) and four cycles of ifosfamide, carboplatin, and etoposide (ICE), with appropriate dose adjustments based on age and tolerance.

Adjuvant therapy was initiated due to the patient’s high-risk pathological features. Postoperative chemotherapy was administered using a regimen of vincristine, actinomycin D, and cyclophosphamide (VAC). The patient experienced only mild bone marrow suppression, with Grades 1–2 neutropenia, and there were no severe chemotherapy-related toxicities. At 5 months of age, radiation therapy commenced to address residual disease, utilizing a two-phase approach. To prioritize organ preservation in this infant, PBT was chosen. The key parameters of the proton beam therapy (PBT) treatment plan are summarized in **Table 3**. The resulting dose distribution is illustrated in **Figure 3**.

**Table 3:** Proton beam therapy treatment plan details.

Feature/parameter	Proton beam therapy (PBT)
Treatment field	Abdominal–pelvic tumor bed (CTVtb)
Total planned dose	14 Gy in 10 fractions (1.4 Gy/f)
Main adjustment reason	To further reduce dose to the right kidney and gastrointestinal tract following initial photon-based radiotherapy
Spinal cord dose limit (Dmax)	<15 Gy
Small intestine dose limit (Dmax)	<16 Gy
Right kidney dose limit (Dmean)	<1.5 Gy
Esophagus dose limit (Dmax)	<15 Gy
Stomach dose limit (Dmax)	<16 Gy
Liver dose limit	Not specifically listed
Positioning and immobilization	Supine, vacuum cushion
Rationale	Eliminate exit dose to further spare organs at risk in an infant
Observed toxicities	Continued mild skin pigmentation; no new major acute toxicities reported
Outcome at end	Completed planned PBT; treatment well tolerated

The dose distribution, demonstrating effective coverage of the target volume while sparing surrounding organs, is illustrated in **Figure 3**.

## Follow-up and outcomes

**Figure 2:**
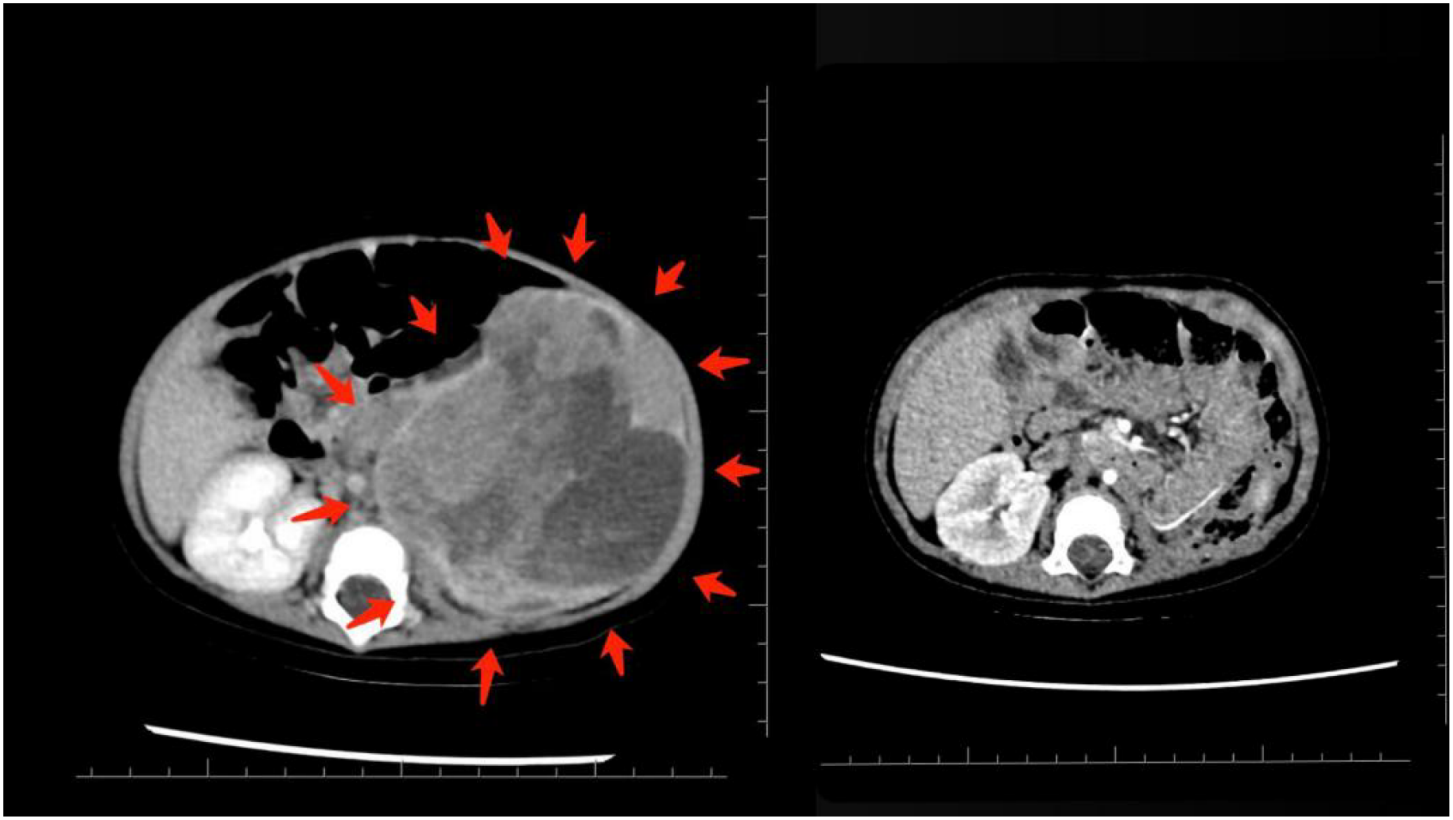
Contrast-enhanced CT scans of the abdomen. Right panel: preoperative imaging demonstrating large heterogeneous masses involving the left kidney and adrenal region. Left panel: postoperative scan after left nephrectomy showing no contrast-enhancing residual mass in the surgical bed.

At 1 year post-treatment, both renal and skeletal outcomes were carefully evaluated. Because the patient underwent a left nephrectomy, preserving the contralateral (right) kidney was paramount. A functional renal mass is critical for long-term protection against renal insufficiency and for supporting overall pediatric growth. Prior studies from the University of Tsukuba have shown that irradiated kidneys in children often demonstrate dose-dependent atrophy, whereas contralateral kidneys consistently exhibit compensatory growth (7). These findings highlight the necessity of stringent sparing of the right kidney during radiotherapy planning. At 1 year post-treatment, laboratory evaluations confirmed preserved renal and hepatic function. As detailed in **Table 4**, liver enzymes (ALT and AST), renal markers (blood urea nitrogen and creatinine), and other key parameters remained within normal, age-appropriate ranges throughout the follow-up period, indicating successful protection of the solitary right kidney and minimal hepatic toxicity. Preoperative imaging, shown in **Figure 2**.

**Table 4:** Summary of the trends in key hematological, hepatic, and renal function parameters.

Parameters	Unit	Before PBT	During PBT	After PBT
WBC	(×10^3^/μL)	12.83	5.09	3.67
RBC	(×10^6^/μL)	4.24	3.62	3.45
Hb	g/L	107	90	88
PLT	(×10^3^/μL)	159	216	106
ALT	U/L	17	27	29
AST	U/L	29	51	59
ALP	U/L	276	191	232
γ-GTP	U/L	18	11	16
CRE	μmol/L	17	14	13
BUN	mg/dL	8	11	9
eGFR	mL/min/1.73 m^2^	63.74	82.26	87.34

PBT, proton beam therapy.

**Figure 3:**
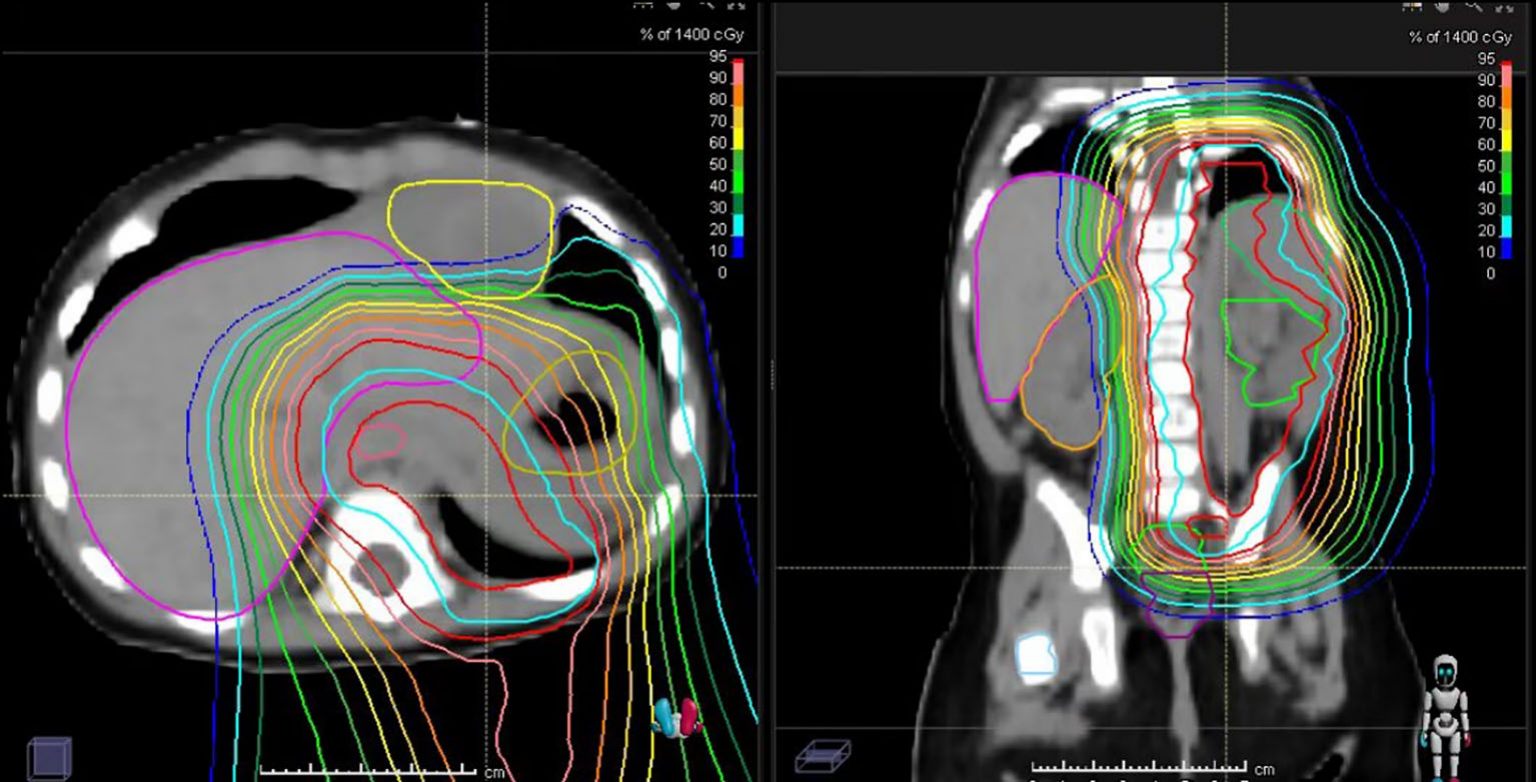
Proton beam therapy (PBT) dose distribution for the abdominal–pelvic tumor bed.

Growth and musculoskeletal development were also closely monitored. Radiotherapy on the spine and pelvis carries a well-documented risk of vertebral growth inhibition, asymmetry, or scoliosis. A Tsukuba University study analyzing 353 vertebral bodies in 23 pediatric patients treated with PBT demonstrated clear dose-dependent suppression of vertebral growth, even at low doses (8). This underscores the importance of minimizing vertebral irradiation in treatment planning.

**Figure 4:**
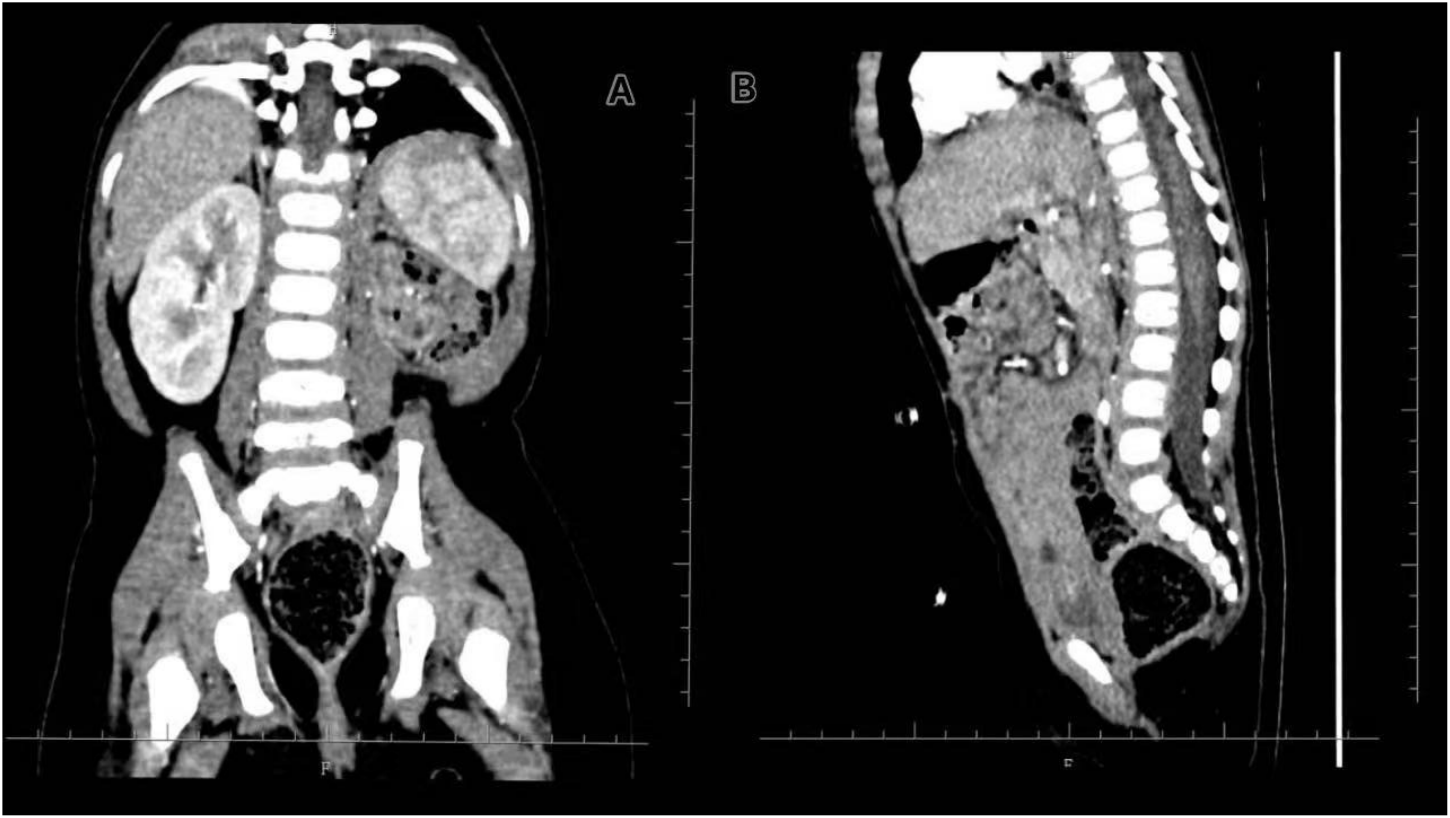
Follow-up CT to assess vertebral body growth after proton beam therapy (PBT). **(A)** Coronal reconstruction demonstrating spinal alignment and symmetry. No significant scoliosis or vertebral wedging is observed. **(B)** Sagittal reconstruction demonstrating vertebral height and spinal curvature.

At the 1-year follow-up, her growth and musculoskeletal development were carefully assessed. She was 5 months old at the start of treatment (height 65 cm, weight 6 kg). At 18 months, her height was 75 cm and weight was 9.6 kg, corresponding to mild growth retardation but with preserved nutritional status and overall health. She achieved appropriate motor milestones (standing, crawling, and independent walking between 12 and 15 months) and demonstrated age-appropriate cognitive and language development with only a mild delay. On physical examination, there was mild asymmetry in the left flank and lumbar region within the irradiated field, but no significant scoliosis, limb length discrepancy, or gait disturbance. Representative follow-up imaging confirming the absence of significant vertebral body growth disturbance or scoliosis is provided in **Figure 4**. Given the radiation exposure to portions of the spine and pelvis, periodic monitoring is planned to detect late skeletal sequelae such as scoliosis or growth-plate disturbance.

## Discussion

Malignant rhabdoid tumor is an extremely rare neoplasm, and no established treatment protocol exists. Multimodal therapy combining surgery, chemotherapy, and radiotherapy is typically considered. Notably, the literature emphasizes that early diagnosis and multidisciplinary treatment are crucial for improving the survival of patients with this disease (1).

Pediatric cancers are inherently rare, and synchronous multiple malignancies in children pose additional diagnostic and therapeutic challenges. As highlighted in the Children’s Oncology Group review on rare tumors (2), their extremely low incidence makes early clinical suspicion difficult, and the nonspecific nature of initial symptoms often leads to diagnostic delay. Radiologic findings are generally limited, and definitive histopathological diagnosis requires advanced techniques such as immunohistochemistry and molecular testing. However, the scarcity of specialized pediatric pathologists frequently necessitates external expert review. Therapeutically, the absence of large-scale clinical trials hampers the establishment of standardized treatment protocols, resulting in substantial variation in management across institutions. In resource-limited settings, restricted access to high-quality imaging, molecular diagnostics, and advanced modalities such as proton beam therapy may further contribute to delays and reduced treatment intensity.

A malignant rhabdoid tumor (MRT) most often occurs in infancy (mean age at diagnosis: ~11 months) and carries a dismal prognosis. In a population-based analysis of MRT, the annual incidence was estimated at only 30–50 cases in the United States (and fewer than 10 in Japan). Patients who did not receive radiotherapy had a 5-year overall survival of only ~33% (9). Advanced-stage MRT is particularly difficult to control. Even with intensive therapy, reported survival rates at 1–2 years are under 30%–40% for Stages III–IV disease (10, 11). Consequently, an aggressive local treatment approach, complete surgical resection when possible, coupled with multi-agent chemotherapy and adjuvant radiotherapy, is internationally recommended for MRT to improve local control and survival (9–11).

The dual malignancy required careful prioritization of treatment. Given MRT’s aggressive nature and high potential for early recurrence, coupled with the fact that the irradiation field for MRT (10.8 Gy plus a boost to the residual tumor) also covers adrenal-origin neuroblastoma, the multidisciplinary team prioritized the administration of radiotherapy. This strategic choice was based on the understanding that delaying or omitting radiotherapy increases the risk of early recurrence and tumor-related mortality. Thus, postoperative chemotherapy targeting neuroblastoma was temporarily postponed, allowing for early radiotherapy to eliminate any residual MRT cells. Meanwhile, systemic therapy continued to address the neuroblastoma, as infant cases identified as intermediate-risk can often be effectively treated with chemotherapy alone.

Neuroblastoma, one of the most prevalent solid tumors in children, originates from neural crest cells of the adrenal medulla or sympathetic ganglia. It typically presents before the age of 5, with approximately 650 new cases reported annually in the United States and approximately 200 in Japan (12, 13). The prognosis for neuroblastoma varies based on multiple biological factors, including the age at diagnosis, disease stage, and MYCN oncogene amplification status. Infants without MYCN amplification usually fall into the intermediate-risk category, exhibiting a favorable prognosis, with 5-year survival rates exceeding 90% through chemotherapy and surgery alone, often excluding the need for radiotherapy (14). In contrast, high-risk neuroblastoma, characterized by features such as MYCN amplification, tends to have a significantly poorer outcome. Omitting radiotherapy in high-risk cases has been linked to higher rates of locoregional relapse and worse overall survival (15). Consequently, current treatment protocols typically incorporate radiotherapy at the primary tumor site for high-risk neuroblastoma as part of a multimodal therapeutic approach.

Evidence regarding prenatal or *in utero* risk factors for malignant rhabdoid tumors is extremely limited. To date, no consistent maternal exposures, environmental agents, or perinatal conditions have been reproducibly linked to rhabdoid tumor risk. Most cases appear to result from early developmental genetic events, either *de novo* somatic SMARCB1/SMARCA4 alterations (16) or, less commonly, germline mutations associated with rhabdoid tumor predisposition syndromes, which likely arise during embryogenesis rather than from modifiable prenatal exposures (17). Because these tumors typically present in infancy, current epidemiologic data do not support any clearly defined prenatal risk factor, although the literature remains sparse.

Notably, there is a lack of large registry data or comprehensive reviews documenting the synchronous occurrence of MRT and adrenal neuroblastoma during infancy. Consequently, the coexistence of these two aggressive malignancies in a single patient underscores an extraordinary clinical scenario devoid of established treatment protocols.

**Figure 5:**
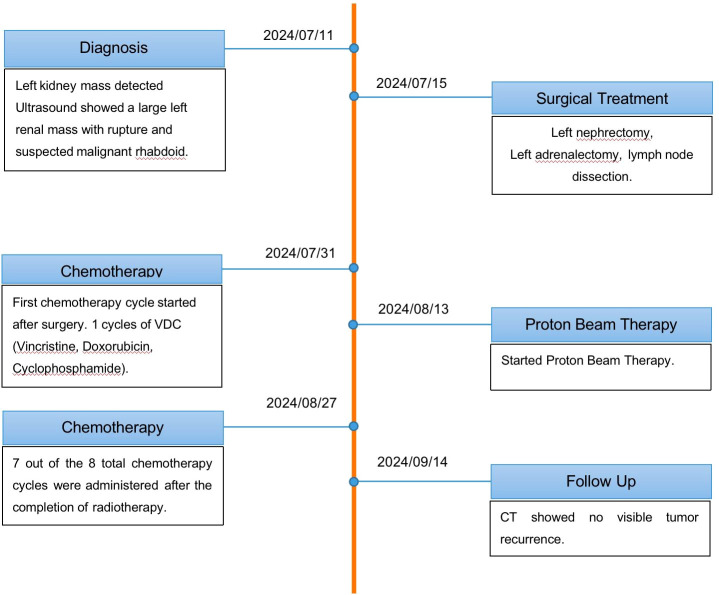
Treatment timetable.

Given the stark contrast in prognosis and treatment requirements between these two malignancies, the multidisciplinary team faced a critical decision regarding treatment sequencing. The treatment course is summarized in **Figure 5** (Treatment Timeline). Collectively, studies on PBT for abdominal tumors have demonstrated excellent local control and significant organ sparing. Hill-Kayser et al. and Hattangadi et al. reported that in a cohort of high-risk pediatric neuroblastoma patients, PBT resulted in zero local failures and significantly reduced radiation dose to the kidneys, lungs, and heart compared with conventional photon therapy, confirming that PBT is particularly advantageous when tumors are located near critical abdominal organs (18, 19). Lim further showed that pencil-beam scanning PBT with motion mitigation strategies is clinically feasible for abdominal neuroblastomas in children, reinforcing that this technique is an appropriate technological choice in very young patients (20). In a comparative planning study, Hill-Kayser et al. (2013) also found that proton therapy markedly spared the contralateral kidney compared with conventional photon therapy, with no observed local recurrences, thereby strengthening the justification for proton use (21). For the rare context of synchronous tumors, Shao described two infants presenting with concurrent neuroblastoma and hepatoblastoma, emphasizing both the rarity of multi-primary presentations and the importance of adopting organ-sparing strategies in such complex cases (22). Finally, within the rhabdoid tumor spectrum, McGovern reported outcomes of children with Central Nervous System (CNS) atypical teratoid/rhabdoid tumor treated with proton therapy, showing effective tumor control while reducing long-term toxicities, thus supporting the application of proton therapy in rhabdoid predisposition settings (23). This case represents a highly unusual synchronous presentation of MRT with neuroblastoma, for which no established guidelines exist. A population-based analysis of pediatric renal rhabdoid tumors, drawing from the California Cancer Registry, reported an annual incidence of 0.19 per 1 million children under age 15, highlighting the extreme rarity of this malignancy (24). Meanwhile, neuroblastoma, although more common, has an annual incidence of approximately 10.5 per 1 million children under 15 years of age. The incidence is highest in infancy, with approximately 65 cases per 1 million live births in the first year of life, according to U.S. registry data (25). However, no large registry or comprehensive review has documented synchronous presentation of MRT and adrenal neuroblastoma in infancy. Thus, the occurrence of these two aggressive malignancies in a single patient underscores an extraordinarily unusual clinical scenario with no established treatment guidelines.

Delivering radiotherapy to a 5-month-old infant posed considerable challenges, particularly with respect to minimizing long-term adverse effects. To address this, we maximized the dosimetric advantages of PBT, which allowed us to sculpt the dose while sparing critical developing organs. Reports of proton therapy in infants under 6 months old are scarce. This case demonstrates the feasibility of this approach under carefully controlled circumstances. The potential for serious late effects from radiation in infants is well documented. Paulino et al. reported that among infants (<1 year old) treated with radiotherapy for neuroblastoma, the cumulative incidence of musculoskeletal toxicity was 38.5% at 10 years and 47.3% at 15 years post-treatment. Moreover, five of six infants who received radiation before 6 months of age developed significant musculoskeletal growth abnormalities (24). Similarly, Li and colleagues demonstrated a clear dose-dependent inhibition of vertebral body growth after pediatric proton therapy, emphasizing the importance of minimizing exposure to bones and growth plates. Together, these findings underscore the need to carefully balance tumor control with skeletal preservation in very young patients.

It is important to note that despite the absence of MYCN amplification, our patient’s adrenal neuroblastoma exhibited several high-risk features, including undifferentiated histology, a high proliferative index, lymph node metastases, and residual disease. According to the International Neuroblastoma Risk Group classification, such adverse factors can upgrade an infant’s risk stratification even in the absence of MYCN amplification (26). Recent analyses suggest that infants under 18 months old with biologically aggressive disease should be considered high-risk, with 5-year event-free survival under 50% despite favorable age and MYCN status (27). Furthermore, while age under 12 months is traditionally associated with better outcomes, there is evidence that the risk of relapse increases for patients beyond approximately 15 months of age. London et al. demonstrated that event-free survival drops significantly for children older than 460 days (~15 months) at diagnosis (28). Although our patient was only 4 months old at diagnosis, the combination of multiple high-risk features likely confers a substantial risk of treatment failure. This consideration reinforced our decision to pursue an aggressive treatment strategy, including radiotherapy, to maximize the chances of a cure.

## Conclusion

Our experience with this case demonstrates that curative-intent treatment, including radiotherapy, is feasible even in an infant under 6 months old when carefully planned and executed using advanced techniques. The decision to proceed with early radiation was justified by the aggressive biology and high recurrence risk of MRT and was only possible through careful multidisciplinary planning. Multidisciplinary coordination was essential to tailor and time the therapies for each tumor. This case highlights how innovations like proton beam therapy can expand treatment possibilities for the very young pediatric population. Accumulation of further cases and long-term follow-up data will be important to better define the role and safety of high-precision radiotherapy in infants.

## Patient perspective

The child’s parents reported feeling relieved after the successful treatment and grateful for the multidisciplinary care. They were especially concerned about long-term developmental effects but were reassured by the close follow-up.

## Data Availability

The original contributions presented in the study are included in the article/Supplementary Material. Further inquiries can be directed to the corresponding author.
